# Dental Neurasthenia

**Published:** 1901-07-15

**Authors:** W. H. Whitslar

**Affiliations:** Cleveland, Ohio


### DENTAL NEURASTHENIA.

BY W. H. WHITSLAR, M.D., D.D.S., CLEVELAND, OHIO.

Abstract of paper read before the Tri-State Dental Meeting, Indianapolis, June, 1901.

Neurasthenia in dental practice seems more common among women than men. Single men and married women are most afflicted, and the age is between eighteen and fifty-five years.

It is occasionally found in children, but is often unrecognized among them. Persons predisposed to neurasthenia are affected by climate. Dryness of the atmosphere predisposes to nervous excitability, which is an expression of neürasthenia. Dryness causes an absorption of the natural fluids of the body, and, as dry air is a poor conductor of electricity, the body becomes overcharged and the nervous system becomes more susceptible to internal or external excitation. Extremes of cold and heat cause nervousness.

Those who by hereditary or other influences have become neurotics or degenerates, or both, are most susceptible to loss of neuricity. Men and women of genius are liable to neurasthenia because as a rule they may be deficient in mental and physical capacity in some direction.

From numerous definitions I assume that neurasthenia is not exactly a disease in itself, but an incoordinate action of neurility wherein it may be made the basis of many functional diseases. These definitions also indicate that two forces are concerned in neurasthenia: *neurosis* and *psychosis*.

Neurasthenia affects the whole system, and its principal symptoms are *nerve weakness* and *nerve irritability*.

In neurasthenia the special senses are not always affected, but may become exciting causes of the disorder. For instance, neurasthenia in children is often ascribed to "overwork," when it finds its etiology in astigmatism, muscular insufficiency, myopia or hypermetropia.

The far-sighted (hypermetropic) child neglects books and cares for out-of-door sports; the near-sighted (myopic) child does not care to play out of doors and reads more than is usual or best for him; hence we notice children of the latter class acquiring sedentary habits and becoming nervous and irritable. They lose the power of co-ordination and neurasthenia is produced long before its cause is recognized. In this class of dental patients we find aggravated conditions which interfere with adequate operations for the care of the teeth. The same results may be said of those afflicted with muscular insufficiency and hypermetropia. An eminent oculist informs me that he observes more affected with neurasthenia of the hypermetropic class than those afflicted with myopia, because the latter class gives up to their conditions more readily.

A large proportion of people use their brains in the struggle for existence. Brain cells are much like muscle; if overworked, exhaustion is produced; but nerve cells recover if allowed to rest for a sufficient time, the recovery being slow. From the loss of cell substance there must be much waste product circulating in the blood which has a toxic action and tends to paralyze the system. Intoxication from indigestion involves the whole mucous membrane of the alimentary canal, and especially the mouth, in which there is a change of the secretions and thickening of the mucous membrane. Pyorrhea alveolaris is without question an example of an overworked, exhausted peridental membrane whose tissues are intoxicated with the excessive material in the blood currents and intercellular tissue. It is a neurotrophia or impairment of the nutrition of the nerves. Exhaustion of the peridental membrane when it occurs is often confined to one or more teeth, and is the result, first, of some irritation, and secondly, an attempt to relieve the uneasiness produced by the irritation with the pressure against the occluding tooth. With pressure comes exhaustion, "neurasthenia localis," which eventually terminates in the graver tissue destruction. From the presence in the blood of toxic substances as an outcome of profound neurasthenia, from whatever cause primarily, it is but just to argue that all tissues of the mouth which are subject to blood currents may suffer from the toxic condition.

"Anesthesia does not exist in neurasthenia" (Dercum), hence we may expect undue sensitivity of the teeth and tissue of the mouth. The dental pulp readily responds to external stimulation and wrathfully expresses its anger in painful elements. As neurasthenia is not a constructive disease, but a symptom of exhausted energies, the pulp of the tooth, if subjected to continued irritation, dies without the fortifications of secondary dentin, or pulp nodules may be formed. The pulp is easily irritated in neurasthenics and exquisite sensitivity of the dentin is often noticed, many times to the regret of the dentist. Bridge work dependent upon distant anchorages causes strain of the peridental membranes, producing exhaustion of the cellular elements and eventually their destruction. With this the alveolar processes give way, and, being no longer supported in its place, the bridge is lost.

Neurasthenia is one of the symptoms of decay, or, expressed otherwise, decay of teeth is the result of those conditions which predispose to decay and which are concerned in the biochemism of the tissues. Ranney, the neurologist, states that "Premature decay of the teeth has been brought forward as one of the most striking evidences of the steady increase of the constitutional impairment which has followed our present methods of living." Such is this condition that neurologists, generally, recognize decay of the teeth as a sign of depression of the nervous system. Dr. Michaels, of Paris, in his paper read before the International Dental Congress, refers to saliva as an indicator of the decay of the teeth. The essay declares that the tissues and secretions depend upon the blood plasma; a dyscrasic condition may produce local or general manifestations, taking place alternately and successionally and ending in one of two ways, in either hypoacidity or hyperacidity.

In hypoacidity there is a vital over-activity resulting in the contagious diathesis as in tuberculosis, syphilis, etc. In consequence of this state it is evident that nerve exhaustion is easily acquired. Dr. Michaels' experiments show, too, that dental decay is more prevalent in this state, hypoacidity, and an indicator of the same is the relative proportion of ammonia being greater in the saliva than the sulpho-cyanides. A very important item in the essay was that the activity of dental decay is modified by the diathetic period.

Now, in hyperacidity we find a decrease of vital activity, but an increase of acidity of the blood, and an accumulation of acid products which in their turn poison the system.

The conclusion is that there may come a neurasthenic state either from hypoacidity (over-activity) or hyperacidity in which waste products accumulate.

*Treatment*.—In the treatment of neurasthenia there are two elements to consider, the patient and the dentist himself. In both we sometimes find the same conditions, *i. e.*, worry, fear, exhaustion from temperature, deficient respiration, and psychic disturbances.

The dentist is exhausted by continued physical labor and anxiety for the care of those whose nervous systems are exalted and intractable by common measures. He is compelled to reduce psychic disturbances by putting himself *en rapport* with the patient. This signifies a depletion of his own nervous force. The first principle of this treatment is to secure the *passivity* of the patient. Rest is the *sine qua non* treatment for neurasthenia. In the performance of dental operations there is no opportunity to follow in detail the rest cure, for the lack of time. We first produce, as above stated, passivity; then comes *faith*. With a basis of passive or restful thoughts and the concurrence of faith or confidence in the dentist, he (the dentist) now is able to suggest orally, or through the subjective mind, his control of the sensations of the body.

If there is any condition that requires care it is at this period, for many cases of neurasthenia drag out for years and the patient eventually dies of some intercurrent disease. As shock is often one of the causes of this disorder, all subsequent operations are under restraint and should be in many cases only temporizing. It is advisable to prepare the patient for an operation by suggesting a period of rest previous to the visit. A good night's rest, by the use of medicaments, as a sedative or a stimulant, as the case requires, is frequently desirable. Strychnia, bromide of potassum, antipyrine, morphine (used rarely), are some of the most reliable remedies. Hot water as a drink before and after operations is one of the most valuable remedies, and its value may be enhanced by the use of some of the milk foods of which we have familiar knowledge. Good food is at all times essential to the well-being of any individual, and it has double value in neurasthenia. Good sleep, good food, are health restorers.

In local neurasthenia of parts of the body, for such may occur, we believe, the conditions of the part suggest the treatment. It must be remembered that there are different phases of neurasthenia and a specific treatment is not known.

DISCUSSION.

Dr. E. T. Loeffler, of Saginaw, Mich.: Neurasthenia is a subject of great interest to the dentist, as it confronts him in all operations upon the sensitive structures of the mouth. While a disease affecting primarily the spinal nervous system, it may involve the entire nervous organism. Neurasthenics seem to be constantly increasing in number, and the more prolonged and taxing operations often required at the hands of the conscientious practitioner can not be made where most needed. It is a timely subject and one that all must understand and be able to treat or at least be able to temporarily control. In order that a dentist may do this successfully we must broaden our conceptions of the dentist's field. It involves the whole subject of normal and impaired nutritive function. Unless we can secure this knowledge we shall not be able to correctly diagnose or treat these conditions. It is another evidence of the ever-increasing burden which modern conditions are putting on the practice of our profession. Our college curriculums must be extended and broadened, and practitioners must prepare to meet these conditions.

Dr. Barnes, of Cleveland, Ohio.—I am interested in the suggestion of the essayist that the dentist himself needs to take treatment for his fatigued nervous system. I have found much benefit in changing my occupation. After a tiresome day's work, or an operation which nervously exhausts me, I find a ride on my bicycle, or a few trips up and down my yard behind the lawn mower will more effectively resuscitate me than going to bed. The habit of going away from home for a few weeks in the summer for recreation may answer for most people. But unless some manual exercise accompanies the vacation there will not be secured adequate results for time and money spent. In my judgment, the dentist will be better able to control himself and influence his patient by regular habits and the habitual indulgence in exercises which will equalize the demands upon all the organs and tissues of the body. For recreation, exercise those muscles and tissues which are not already fatigued; new scenes, new work and recreations which please the mind.

Dr. I. Douglas, Romeo, Mich.: I once had for a patient, a clergyman, who was so nervous that it was difficult to do anything for him. It was found that he had a tape-worm; its removal so cured his neurasthenia that he can have dental operations made without especial difficulty. Children are often made nervous from similar disorders. As a simple and effective remedy for resuscitation from fatigue I use with gratifying results hot water. After a hard day's work, one to three cups of hot water before supper acts like a charm to dispel the feeling of fatigue.

Dr. H. A. Smith, of Cincinnati: Dr. Cassidy tells me that one of the waste products in nerve fatigue or neurasthenia is lactic acid. If this is so to any extent in nervous disorders of the dental tissues, it is no wonder that we have destruction not only of the soft structures but of the teeth also. I would take exceptions to Dr. Barnes' idea of recuperation by change of occupation. It is physiological rest that we need, and this can be better secured by lying prostrate on one's back on a comfortable bed, so that all organs may perform their functions with the least resistance, and consequently most effectively. Walking, riding, wood-chopping and similar diversions may meet some needs, but not mine.

Dr. Booth: I see no reason why we may not use in cases of excessive nervousness some of the less offensive nerve sedatives. I have used with gratifying results chlorotone. It acts very nicely and I have had no undesirable results from its use.

Dr. C. M. Wright, of Cincinnati: Much depends on the nature of the nerve irritation as to line of treatment to be adopted. It may be wholly local or it may be local with systemic manifestations or the reverse, systemic disorders with local manifestations, whichever it may be, the effort nature is making to correct the disorder should receive careful attention, and all efforts to cure should be made so as to harmonize with this effort. It might be to go to bed in one case, and a foot race in another, or it may be that two or three glasses of beer would be of more benefit than the same quantity of hot water.

Dr. J. Taft, of Cincinnati, Ohio: Nerve fatigue seems to have become the normal condition of many people, judging by the ease with which they can be thrown off their balance. It is very questionable if much can be done to overcome this condition by systemic medication. It may be temporarily relieved, and medicines as expedients are valuable. But very often nerve depressants will be positively contra-indicated. If it were possible to get such people to change their methods of living or occupation, so that nature might have a chance to restore the fatigued nerve cells to a normal condition, then we might hope to accomplish something in the treatment of local affections resulting from such conditions.

Dr. N. S. Hoff, of Ann Arbor, Mich.: I was much interested in the statement of the essayist that local diseases like pyorrhea alveolaris were manifestations of systemic neurasthenia. Is it not possible that systemic neurasthenia may have more to do with other local disorders than we think? May it not seriously handicap us in capping pulps, treating alveolar abscesses, as well as complicating many other treatments which we are too much inclined to look upon as amenable to our pet formulary? A dentist recently referred a case of pulp canal treatment for my judgment, which he could not satisfactorily treat by the usual remedies. A scrofulous diathesis I have no doubt accounts for the tardy healing in this case.

Dr. F. W. Sage, of Cincinnati: Dentists treat a more sensitive part of the body than the general surgeon, and too often without resorting to any of the obtundents or anesthetics which the general surgeon always uses, even in more trivial operations. Dentists should make more use of anesthetics. In order that the dentist may serve his patients adequately he should preserve always a well-balanced nerve equilibrium, but at the same time he must have due regard for the condition and well being of his patient. This is not always easy. Much can be accomplished by a judicious use of pain obtundents. But the dentist must go beyond the sensitive tooth and be able to appreciate the actual physical condition of his patient. Timid and nervous patients are deserving of sympathy and should be intelligently handled. And the dentist who is best able to recognize these conditions and most appropriately handle them will be correspondingly successful, providing he shall deport himself otherwise in an orderly manner.

DR. H. L. AMBLER, of Cleveland, Ohio: Neurasthenia might be called Americanitis, but this disorder is not entirely confined to Americans, although very prevalent. Have had some success in administering to patients, just before operating, ten to fifteen drops of Jamaica dogwood as a nervous sedative. Also have had success in placing patients in the most comfortable position possible, and sometimes hypnotism may be used advantageously.

DR. EMORY, Newark, Ohio: What rests one person may tire another. One may become both nervously and muscularly fatigued by remaining long in the same position. Energy does not necessarily tire one; and, as one may obtain a sense of rest by changing the position of the body, so one may secure mental rest by diverting one's thoughts. If angry, whistle or sing. If an irritable and impatient patient can be met with a sweet and unruffled temper, much will be gained. An irritable patient and an angry dentist make a very bad combination. One, at least, should keep cool.

Dr. Whitslar, in closing the discussion, said: Local diseases are not always caused by systemic neurasthenia, but diseases of local origin may be made worse by a general nervous disorder, and systemic diseases often express themselves in localized disorders. It is possible with proper instruments or apparatus to diagnose accurately any variation from normal function in the nerve system. This is scarcely necessary, as nervous disorders will always show themselves in some way. The emotions, fear, jealousy, etc., are common manifestations. Would suggest as a name for the disease known usually as fear of dental operations the term odontopathophobia. Psychotic diseases are amenable to all forms of personal magnetic influences, hypnotism, Christian science, and the like. In the matter of exercising we are liable to over-exercise a part of the body while the rest is not normally nourished. The athlete's teeth are poor and exceedingly sensitive, simply because the pulps are deprived of the normal blood pabulum.

